# Supplementary figures and images for: The implementation of rare events logistic regression to predict the distribution of mesophotic hard corals across the main Hawaiian Islands
Source: PeerJ. 2016 Jul 6;4:e2189. doi: 10.7717/peerj.2189 (PMC4941748; doi:10.7717/peerj.2189)

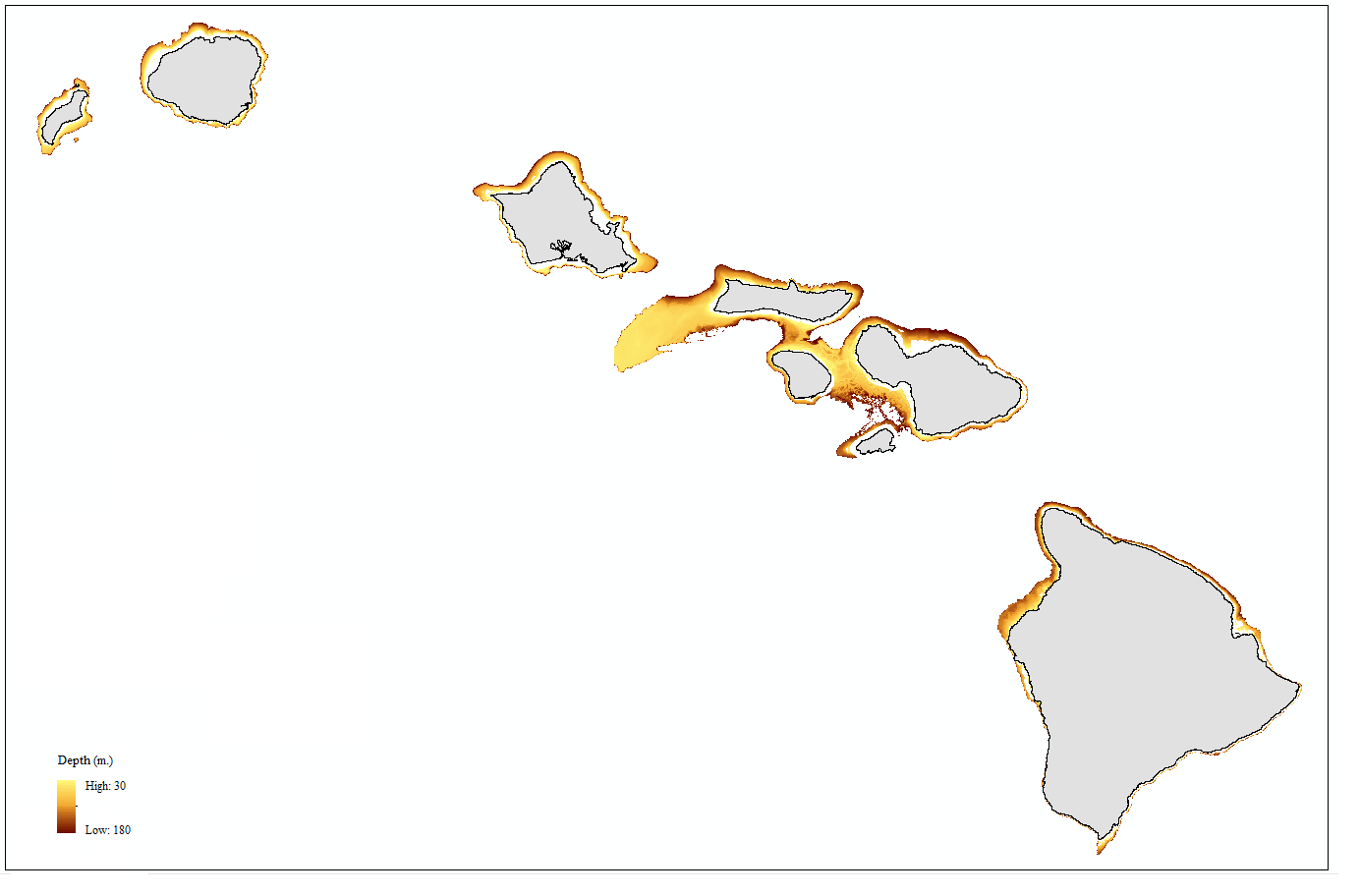

Supplement: Figure S1 [file peerj-04-2189-s005.png]

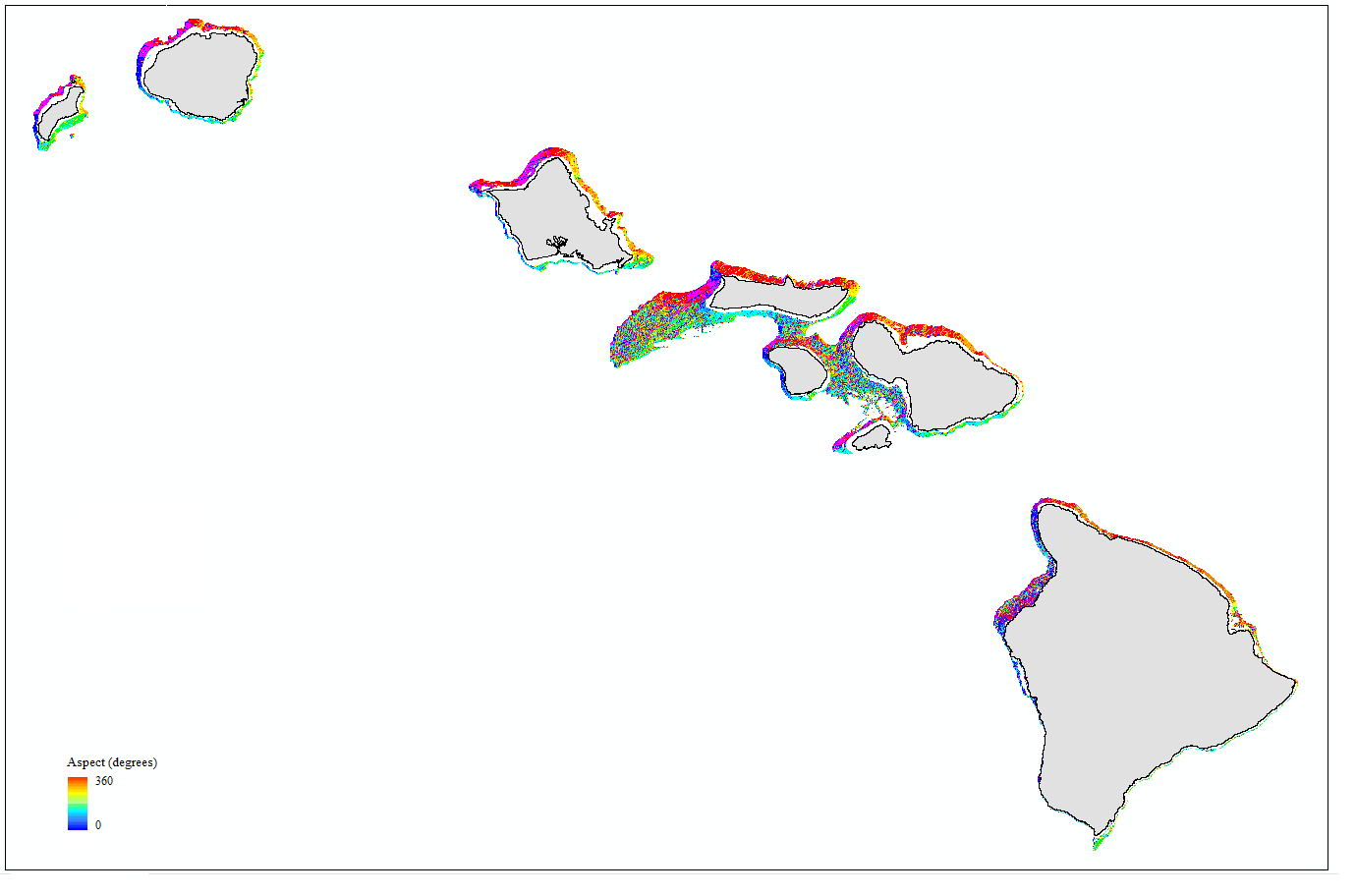

Supplement: Figure S2 [file peerj-04-2189-s006.png]

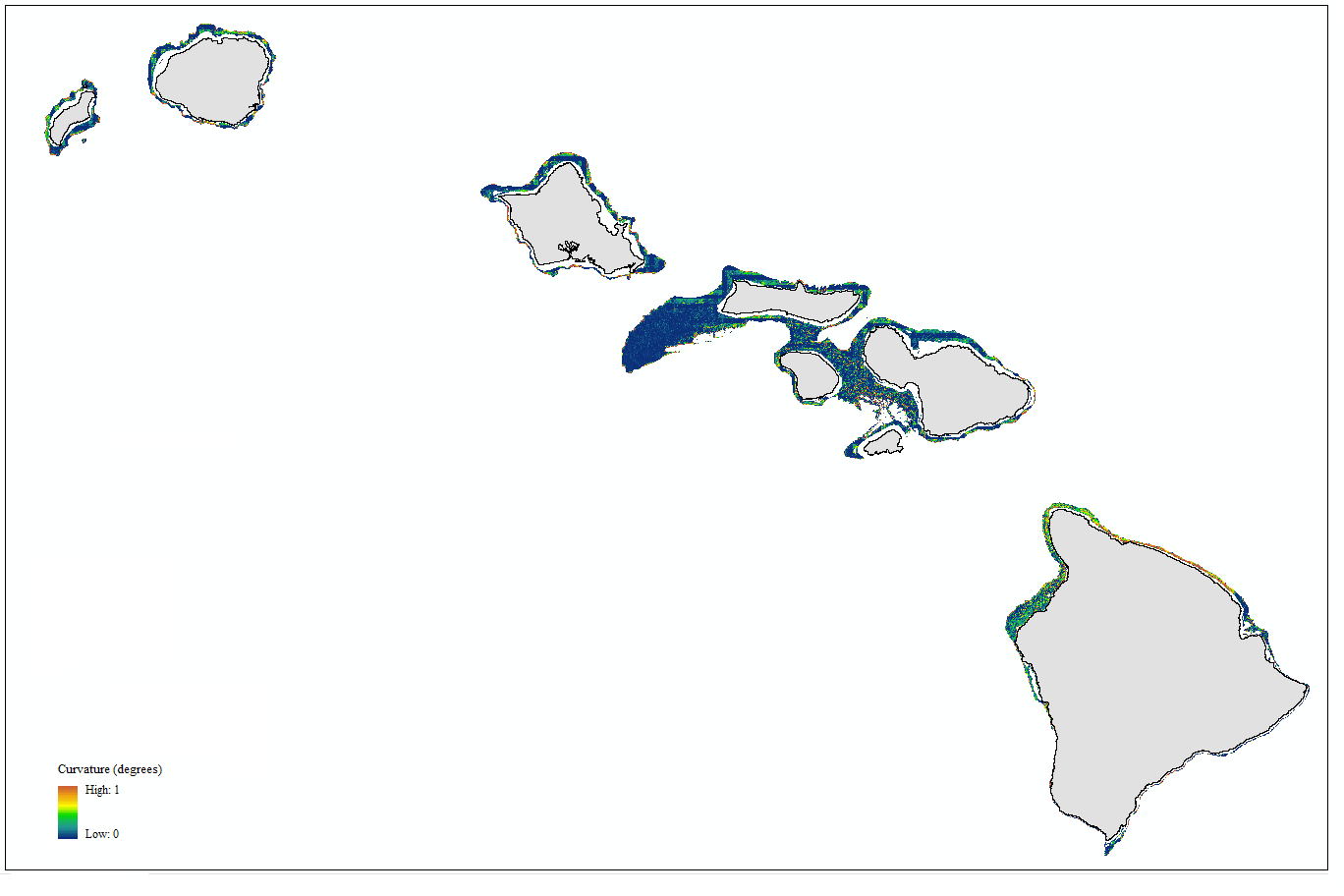

Supplement: Figure S3 [file peerj-04-2189-s007.png]

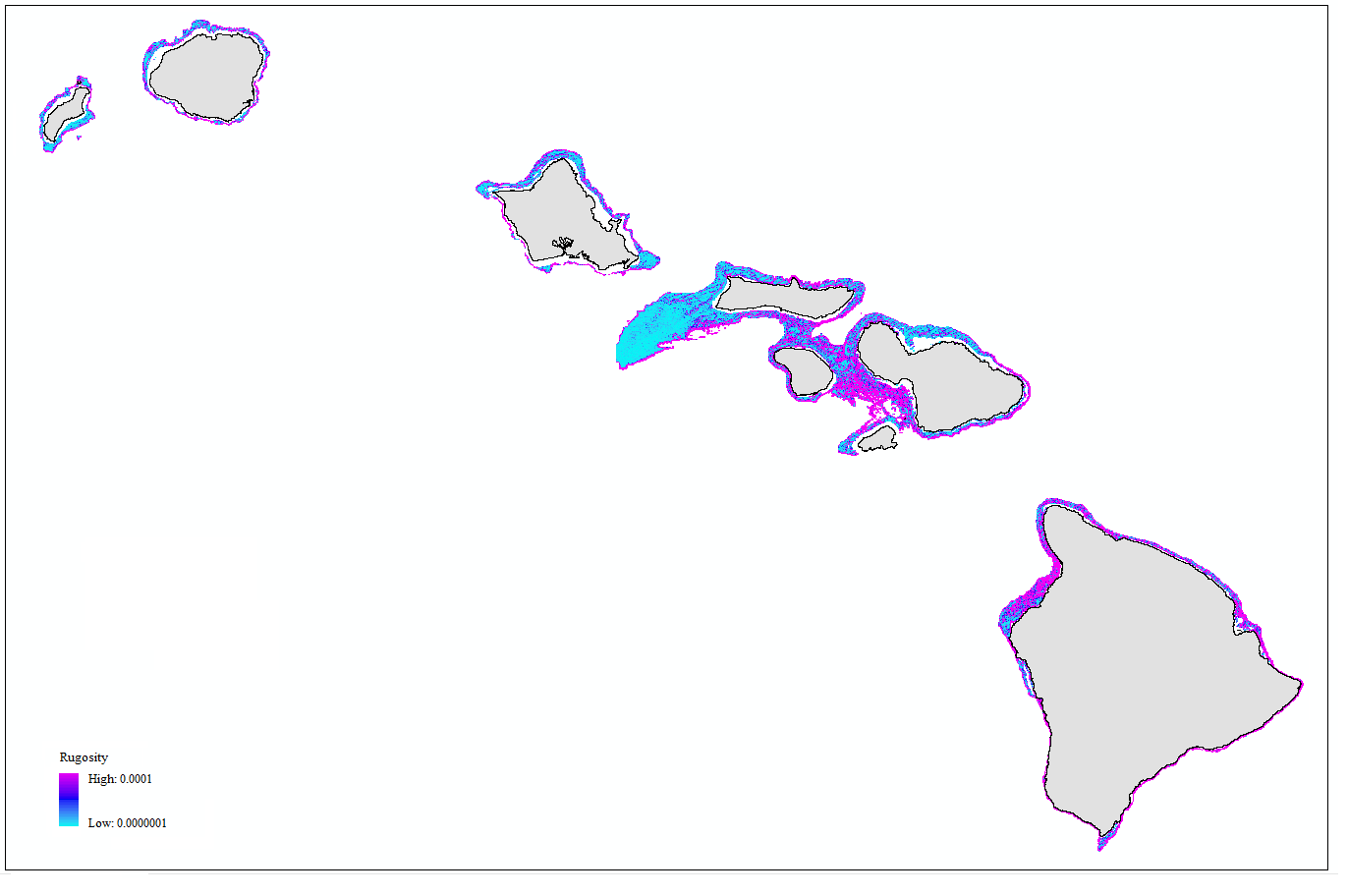

Supplement: Figure S4 [file peerj-04-2189-s008.png]

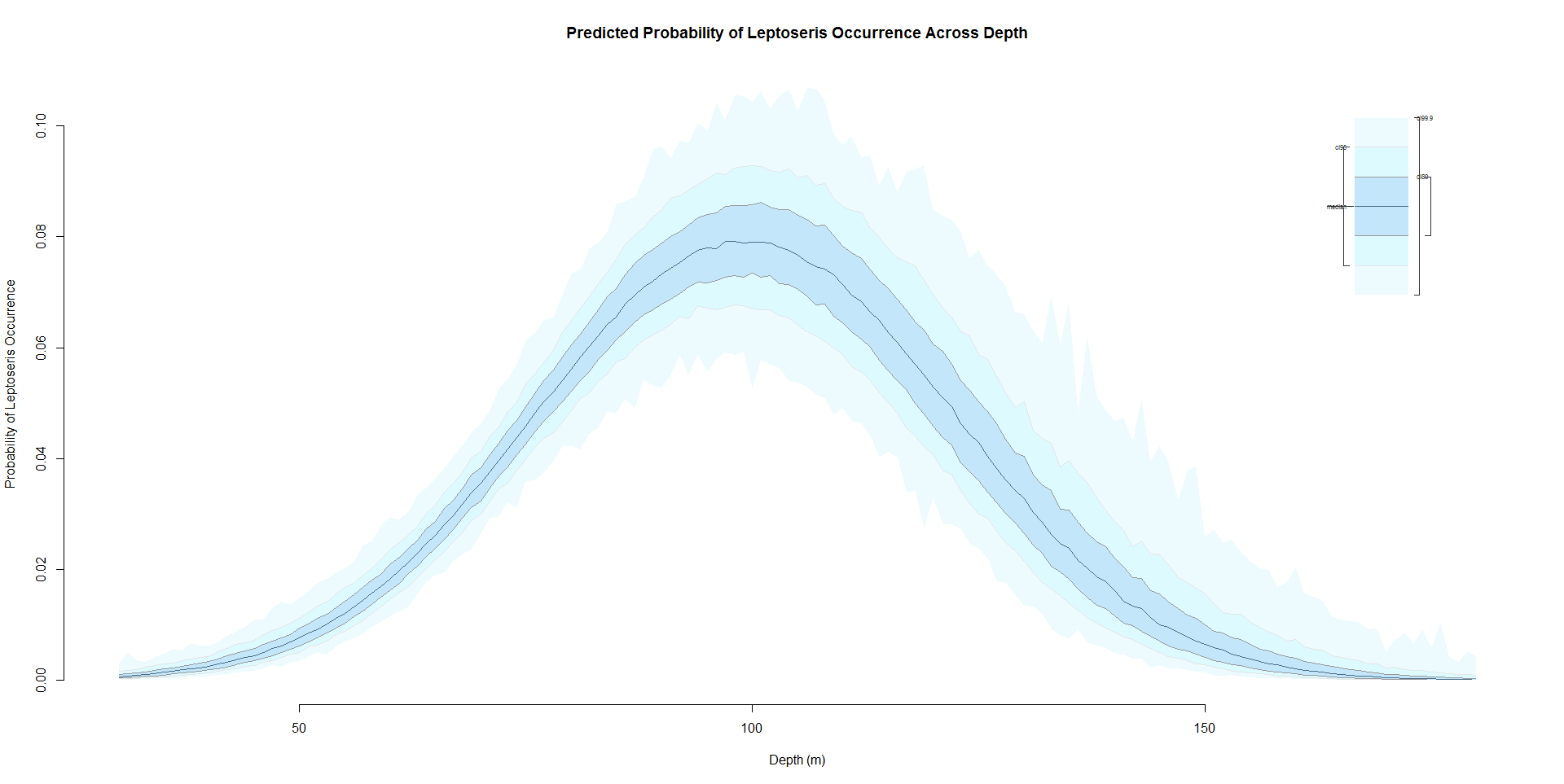

Supplement: Figure S5 [file peerj-04-2189-s009.png]

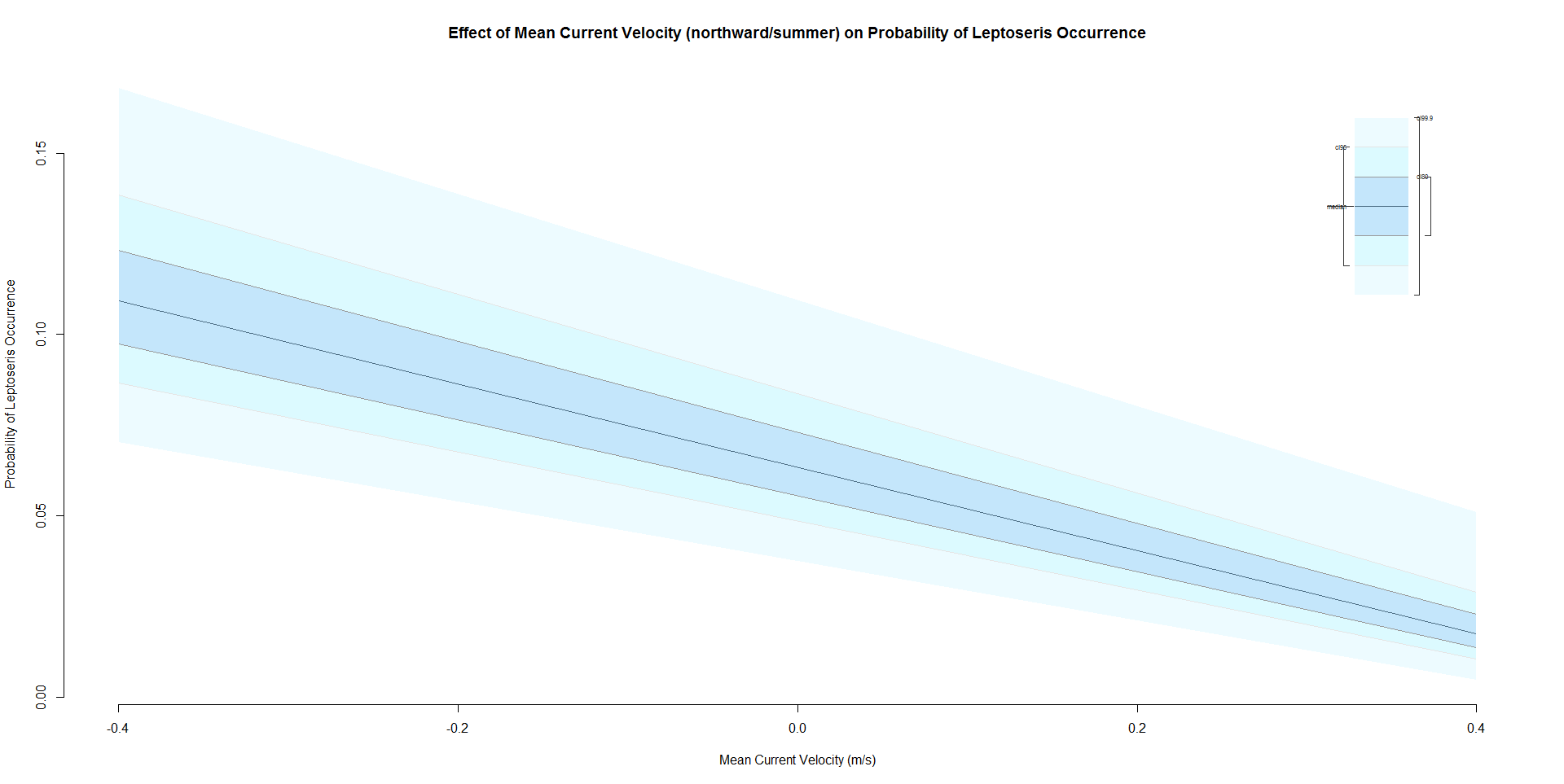

Supplement: Figure S6 [file peerj-04-2189-s010.png]

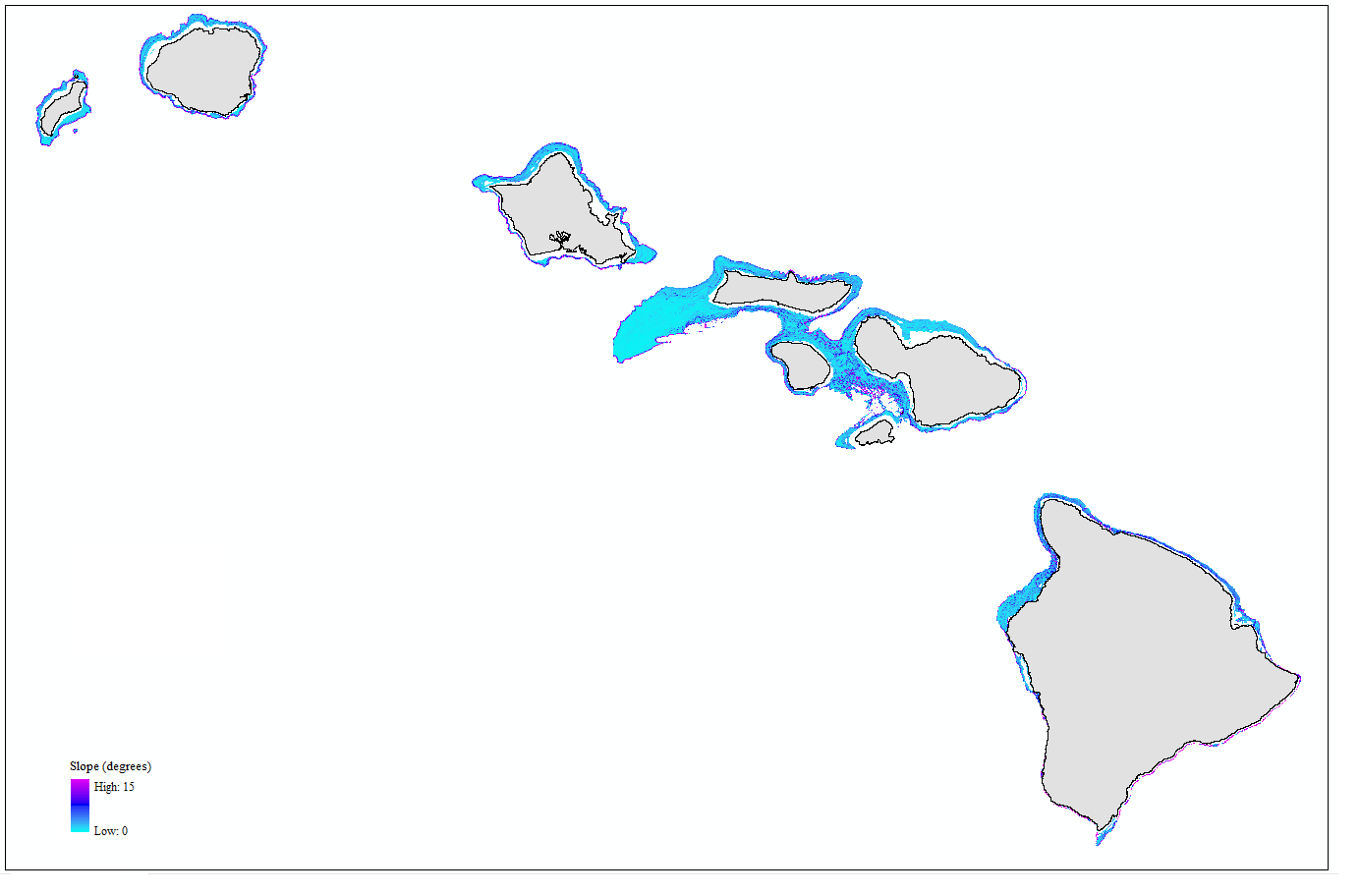

Supplement: Figure S7 [file peerj-04-2189-s011.png]

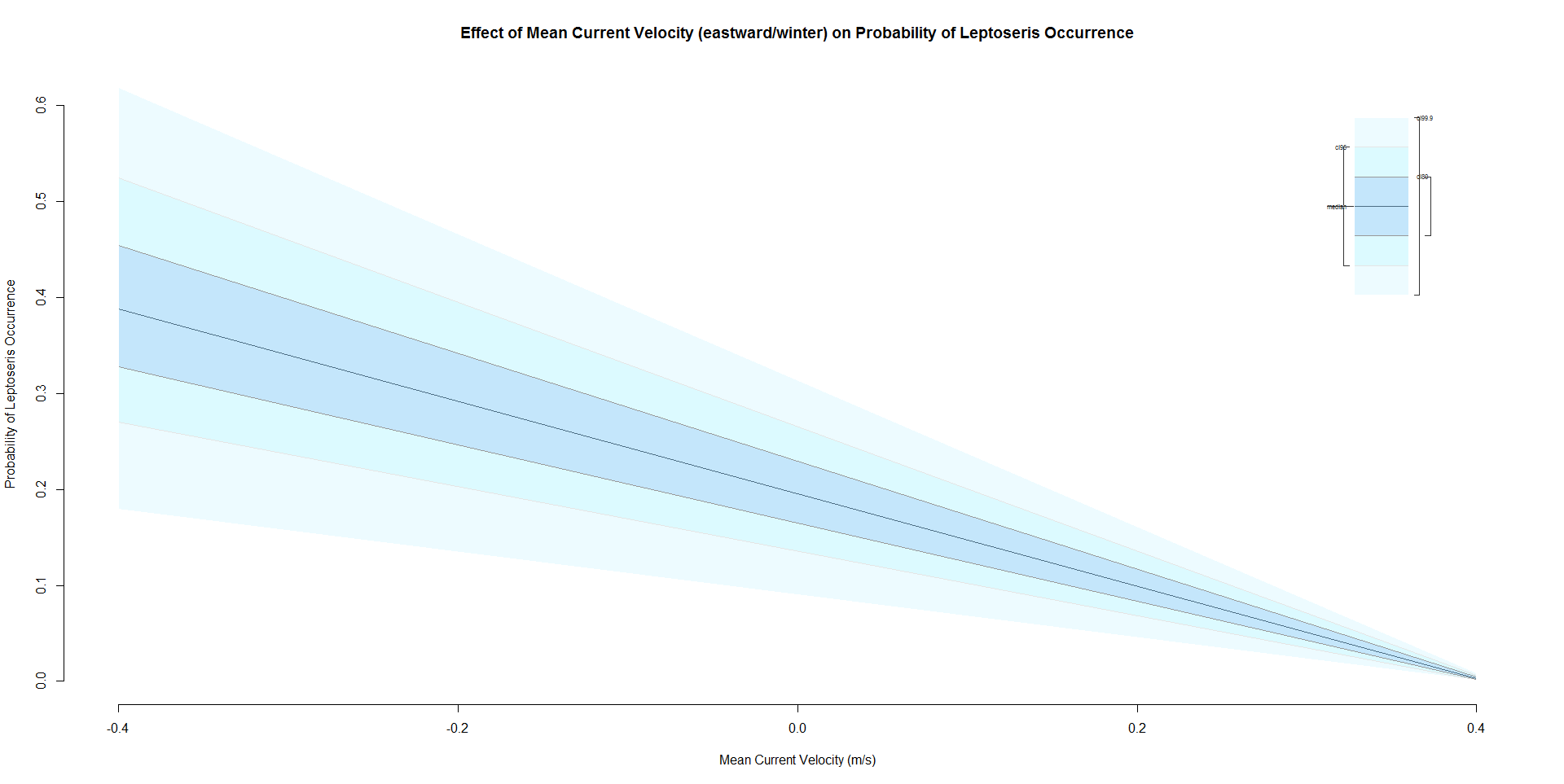

Supplement: Figure S8 [file peerj-04-2189-s012.png]

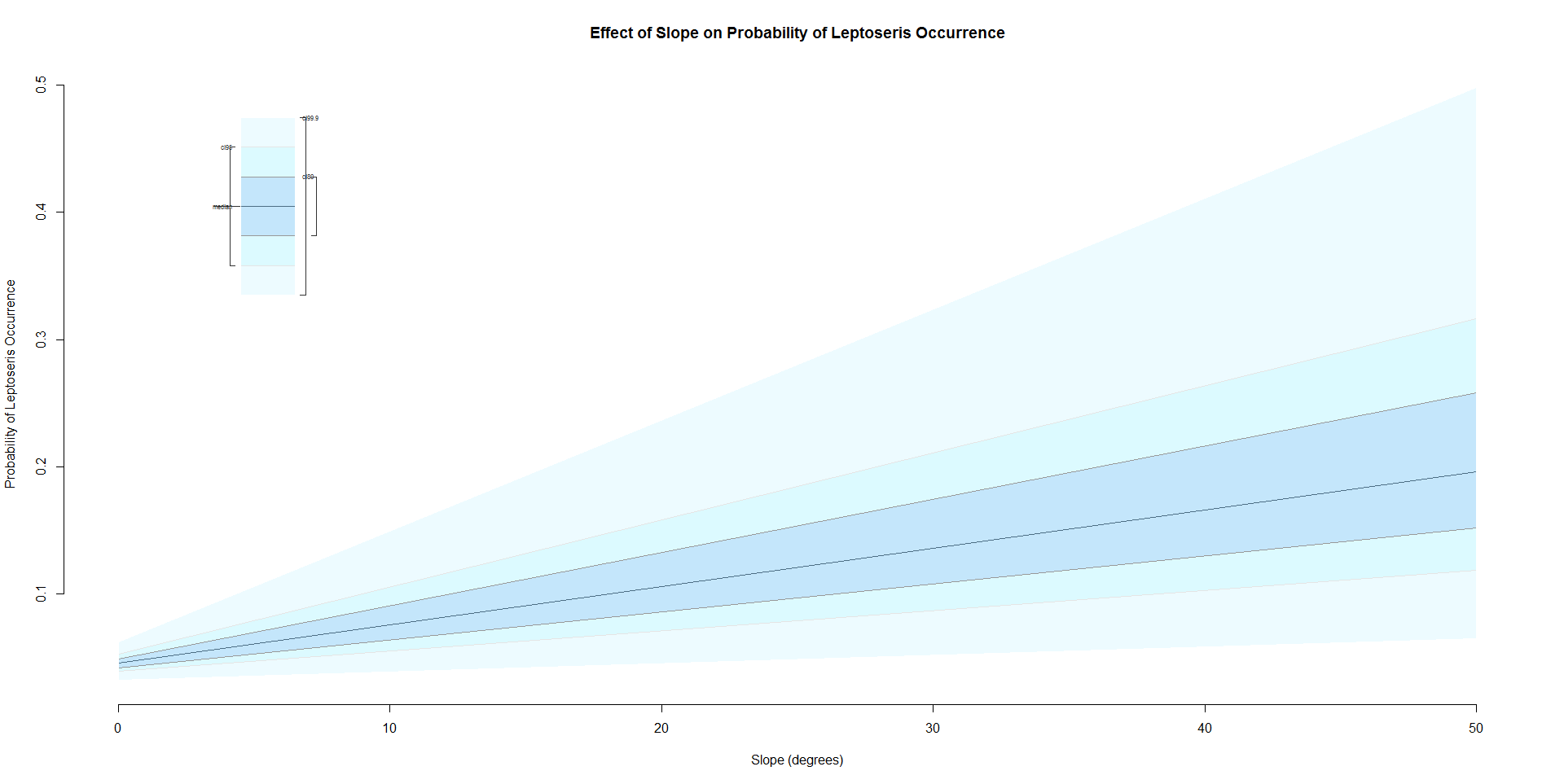

Supplement: Figure S9 [file peerj-04-2189-s013.png]

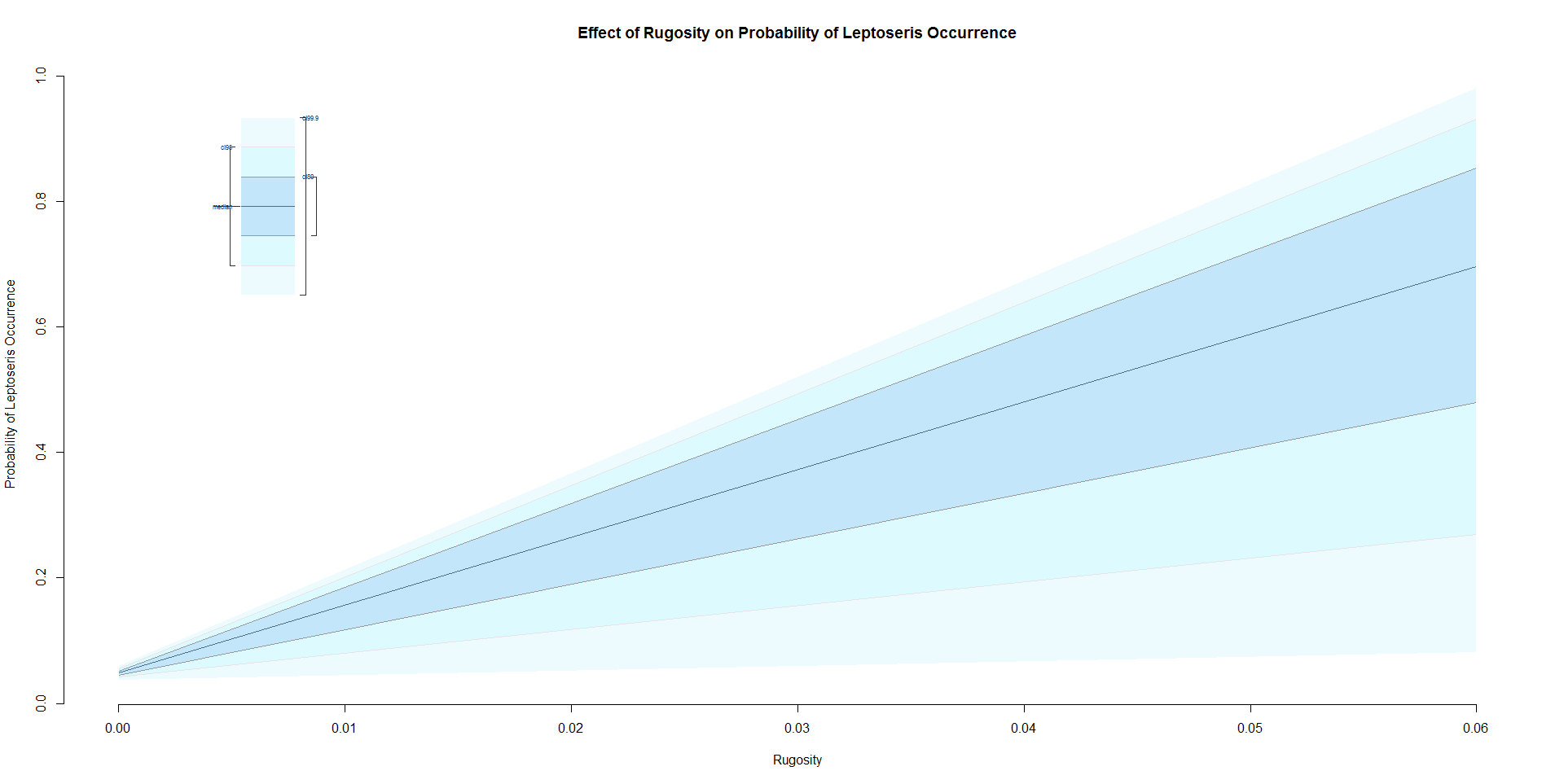

Supplement: Figure S10 [file peerj-04-2189-s014.png]

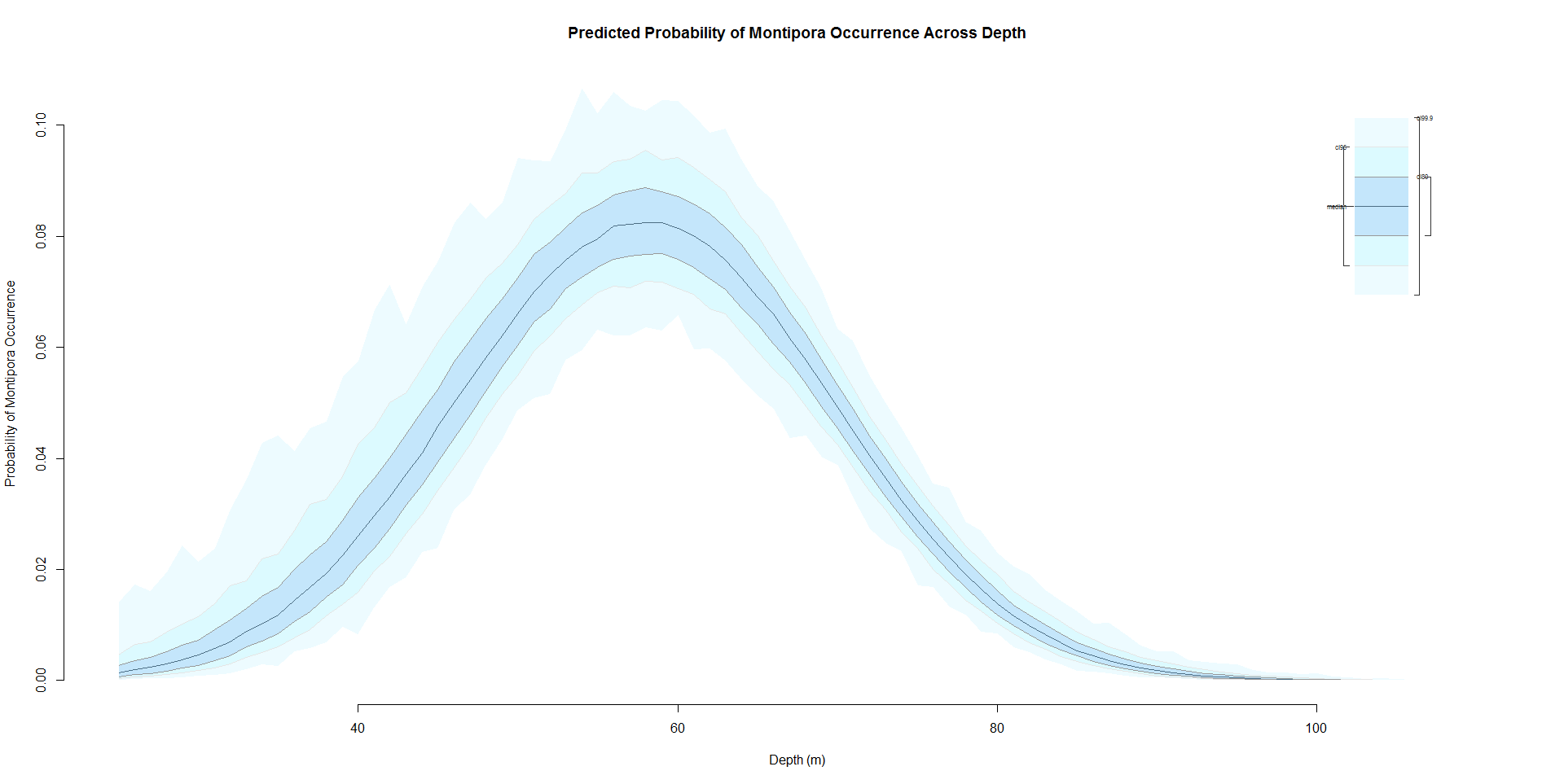

Supplement: Figure S11 [file peerj-04-2189-s015.png]

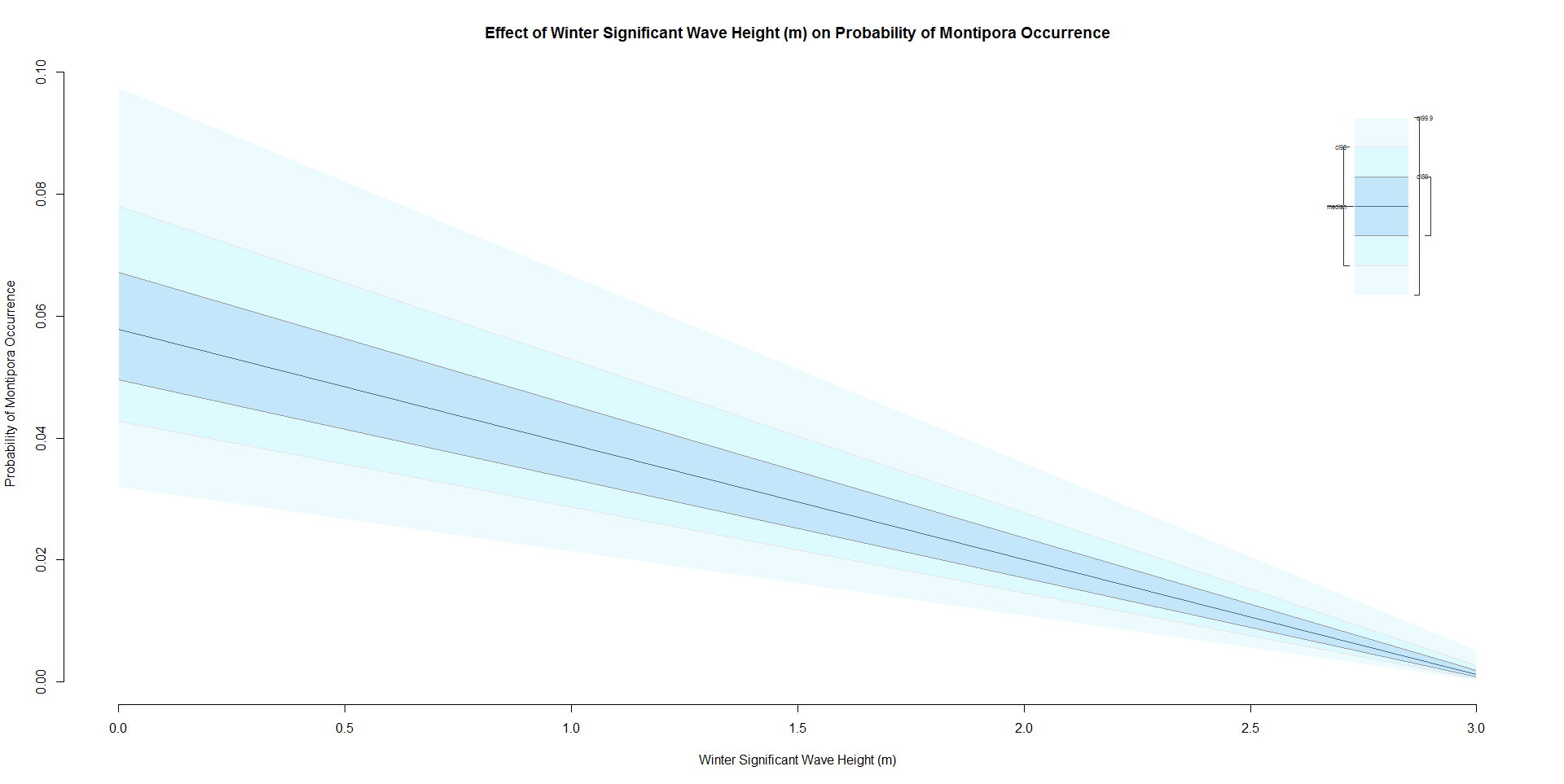

Supplement: Figure S12 [file peerj-04-2189-s016.png]
