# Supplementary material for: The implementation of rare events logistic regression to predict the distribution of mesophotic hard corals across the main Hawaiian Islands
Source: PeerJ. 2016 Jul 6;4:e2189. doi: 10.7717/peerj.2189 (PMC4941748; doi:10.7717/peerj.2189)
Supplement: Table S1 [file peerj-04-2189-s017.docx]

| **Covariate** | **Coefficient estimate** | **Std. error** |
| --- | --- | --- |
| Intercept ($\beta_{0}$) | -12.920 | 1.143 |
| Depth ($\beta_{1}$) | 0.201 | 0.02575 |
| Depth*Depth ($\beta_{2}$) | -0.001 | 0.0001413 |
| Mean current velocity: northward, summer ($\beta_{3}$) | -2.392 | 0.5673 |
| Mean current velocity: eastward, winter ($\beta_{4}$) | -6.625 | 0.8725 |
| Slope ($\beta_{5}$) | 0.033 | 0.008194 |
| Rugosity ($\beta_{6}$) | 64.910 | 18.250 |
