# Supplementary material for: The implementation of rare events logistic regression to predict the distribution of mesophotic hard corals across the main Hawaiian Islands
Source: PeerJ. 2016 Jul 6;4:e2189. doi: 10.7717/peerj.2189 (PMC4941748; doi:10.7717/peerj.2189)
Supplement: Table S4 [file peerj-04-2189-s020.docx]

| **Covariate** | **Coefficient estimate** | **Std. error** |
| --- | --- | --- |
| Intercept ($\beta_{0}$) | -13.990 | 2.158 |
| Depth ($\beta_{1}$) | 0.4407 | 0.06847 |
| Depth*Depth ($\beta_{2}$) | -0.004 | 0.0005433 |
| Significant wave height: winter ($\beta_{3}$) | -1.300 | 0.1925 |
